# Carboxypeptidase E-∆N Promotes Proliferation and Invasion of Pancreatic Cancer Cells via Upregulation of CXCR2 Gene Expression

**DOI:** 10.3390/ijms20225725

**Published:** 2019-11-15

**Authors:** Sangeetha Hareendran, Xuyu Yang, Hong Lou, Lan Xiao, Y. Peng Loh

**Affiliations:** Section on Cellular Neurobiology, *Eunice Kennedy Shriver* National Institute of Child Health and Human Development, National Institutes of Health, Bethesda, MD 20892, USA

**Keywords:** Carboxypeptidase E, pancreatic cancer, cell proliferation, cell invasion

## Abstract

Pancreatic cancer is one of the leading causes of cancer-related mortality worldwide. The molecular basis for the pathogenesis of this disease remains elusive. In this study, we have investigated the role of wild-type Carboxypeptidase E (CPE-WT) and a 40 kDa N-terminal truncated isoform, CPE-ΔN in promoting proliferation and invasion of Panc-1 cells, a pancreatic cancer cell line. Both CPE-WT and CPE-ΔN were expressed in Panc-1 and BXPC-3 pancreatic cancer cells. Immunocytochemical studies revealed that in CPE transfected Panc-1 cells, CPE-ΔN was found primarily in the nucleus, whereas CPE-WT was present exclusively in the cytoplasm as puncta, characteristic of secretory vesicles. Endogenous CPE-WT was secreted into the media. Overexpression of CPE-ΔN in Panc-1 cells resulted in enhancement of proliferation and invasion of these cells, as determined by MTT (3-(4,5-dimethylthiazol-2-yl)-2,5-diphenyltetrazolium bromide) cell proliferation assay and Matrigel invasion assay, respectively. In contrast, the expression of CPE-WT protein at comparable levels to CPE-ΔN in Panc-1 cells resulted in promotion of proliferation but not invasion. Importantly, there was an upregulation of the expression of *CXCR2* mRNA and protein in Panc-1 cells overexpressing CPE-ΔN, and these cells exhibited significant increase in proliferation in a CXCR2-dependent manner. Thus, CPE-ΔN may play an important role in promoting pancreatic cancer growth and malignancy through upregulating the expression of the metastasis-related gene, *CXCR2*.

## 1. Introduction

Pancreatic cancer (PC) is the 12th most common cancer in the world (wcrf.org) and the 7th leading cause of cancer-related death globally [1]. Most PCs are epithelial exocrine cancers with a high occurrence of malignancy. PCs are generally diagnosed in the advanced stages, when metastasis has already occurred, leading to poor prognosis and a high incidence of mortality [2]. The molecular and cellular mechanisms underlying the pathogenesis of PC malignancy are poorly understood. In the early low-grade PanIN-1 (Pancreatic Intraepithelial Neoplasia) stage, *KRAS* is mutated, oncogenic miRNAs are overexpressed, and associated stromal factors are activated. In the PanIN-2 intermediate stage, inactivating mutations in the *p16/CDKN2A* gene and overexpression of *MUC1* are observed. In the late PanIN-3 stage, inactivating mutations in *TP53, BRCA2,* and *SMAD4* genes were found. The tumor environment, especially tumor-stromal interactions, also contribute to the aggressive progression of the disease [1]. Identification of novel molecular factors and mechanisms involved in the development of PC will uncover diagnostic and prognostic biomarkers and therapeutic targets.

Carboxypeptidase E (CPE) is a multifunctional protein. First discovered as a prohormone processing enzyme involved in the synthesis of mature peptide hormones and neuropeptides, it was recently shown to exhibit non-enzymatic functions; acting as a neurotrophic factor contributing to stress-induced neuroprotection and neural stem cell differentiation [3,4]. CPE knock-out mice are obese, diabetic, infertile, and exhibit poor memory and learning. CPE has been shown to be involved in tumorigenesis and cancer progression [5,6,7]. Clinical studies have demonstrated that elevated CPE mRNA and protein levels are correlated with poor prognosis in colorectal [8], hepatocellular carcinoma [9], and cervical cancer patients [10]. 

Wild-type (WT) CPE (53 kDa) has been found in various types of endocrine tumors, including insulinomas [11] and pulmonary neuroendocrine tumors [12], as well as in epithelial-derived hepatomas [13] and gliomas [14]. Secreted CPE-WT promotes proliferation in glioma cells but is associated with anti-invasion activity in these cells [15] and the HT-1080 fibrosarcoma cell line [7]. A CPE mRNA splice variant encoding a 40 kDa CPE-ΔN isoform has recently been cloned from hepatocellular carcinoma (HCC) cells and shown to be 1.7 kb in size. Overexpression of the 40 kDa CPE-ΔN in HCC cells upregulated the expression of metastasis-related genes, including chemokine receptor CXCR2, which is associated with PC malignancy [16,17,18,19]. Elevated tumor expression of CPE-ΔN protein has been correlated with poor prognosis in lung adenocarcinoma patients [20]. Additionally, overexpression of a 46 kDa CPE-ΔN isoform in osteosarcoma (OS) cells resulted in enhanced cell growth, migration, and invasion [21]. Thus, CPE-ΔN variants play important roles in tumorigenesis.

Here, we investigated whether CPE-WT and 40 kDa CPE-ΔN isoform are expressed in Panc-1 and BXPC-3 pancreatic cancer cell lines. A previous study showed that suppression of endogenous CPE in BXPC-3 cells downregulated the growth and chemosensitivity of these cells in vitro and inhibited PC tumor growth in xenograft mouse models [22]. However, the study did not investigate the possible differential regulation of PC development by the CPE-WT and CPE-ΔN variant. Using Panc-1 as an in vitro model of PC, we determined the subcellular distribution of CPE-WT and CPE-ΔN in the PC cells and carried out gain-of-function studies to compare the efficacy of 40 kDa CPE-ΔN versus CPE-WT protein in promoting proliferation and invasion. Finally, we investigated if a downstream target protein CXCR2 [19], that is known to support tumorigenesis and metastasis of PC, is upregulated by 40 kDa CPE-ΔN and whether it mediates the CPE-ΔN induced increase in proliferation of Panc-1 cells.

## 2. Results

### 2.1. CPE Transcripts and Proteins Expressed in Human Pancreatic Cancer Cell Lines

**Figure 1:**
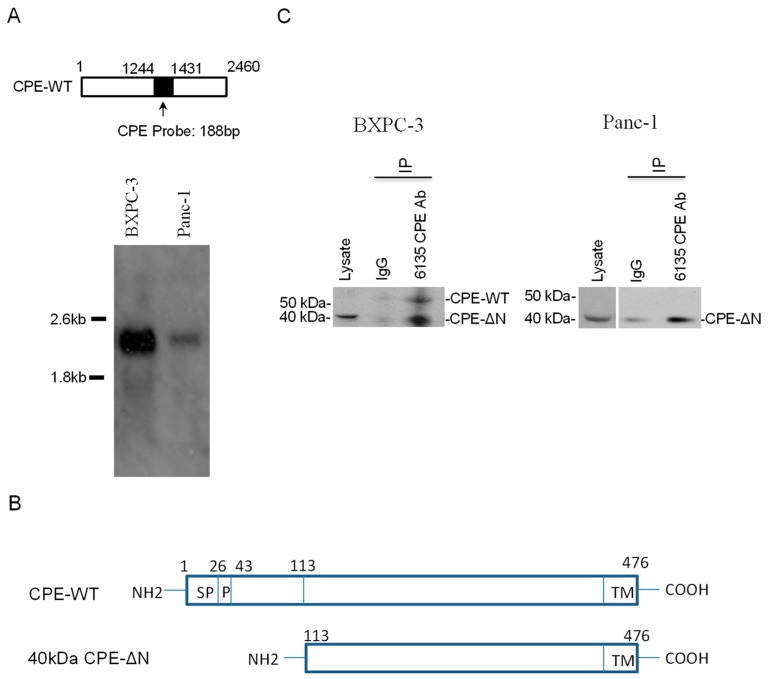
Expression of the carboxypeptidase E (CPE) variant transcripts and proteins in human pancreatic cancer cells. (**A**) Upper panel: Schematic representation of the DIG-labeled CPE probe (shaded box). The numbers refer to the probe covering the region in human CPE mRNA. Lower panel: Northern Blot showing CPE transcripts detected in BXPC-3 and Panc-1. (**B**) Schematic representation of the alignment of deduced amino acid sequences of homo sapiens CPE NM001873.2, CPE-WT, and its variant 40 kDa CPE-ΔN; the 40 kDa CPE-ΔN lacks 112 aa at the N-terminus. Numbers refer to the amino acid positions in CPE protein. SP, signal peptide; P, propeptide; TM, transmembrane domain. (**C**) Western blot using anti-CPE mouse monoclonal antibody showing the expression of endogenous CPE-WT and/or CPE-∆N in BXPC-3 (left panel) and Panc-1 (right panel) cell lysate immunoprecipitated with either rabbit polyclonal anti-CPE specific antibody (6135) or rabbit IgG as control. Non-immunoprecipitated cell lysate of Panc-1 was present in the same blot, separated by two lanes, and developed with longer exposure. Full-length blots are presented in Supplementary Figure S1.

The CPE mRNA expression pattern for two pancreatic cancer lines BXPC-3 and Panc-1 was examined by Northern blot. Two mRNA transcripts, a ~2.4 kb CPE and ~1.7 kb CPE transcript variant, were detected in the BXPC-3 cells (Figure 1A). The 1.7 kb RNA represented a CPE Δ189–386 splice variant encoding a 40 kDa N-terminal truncated CPE protein (CPE-ΔN) and the 2.4 kb, CPE-WT transcript [19] (Figure 1B). In the Panc-1 cells, only a 2.4 kb CPE-WT transcript was detectable. To determine the protein expression of CPE, BXPC-3 and Panc-1 cell extracts were immunoprecipitated with CPE antibody 6135 followed by Western blot using BD monoclonal antibody against CPE. The pull-down experiments revealed expression of a major 40 kDa CPE-ΔN protein and a minor 53 kDa CPE-WT protein in BXPC-3 (Figure 1C, left panel) and a 40 kDa protein in the Panc-1 cells (Figure 1C, right panel).

### 2.2. Subcellular Distribution of CPE and CPE-ΔN in Panc-1 Cells

**Figure 2:**
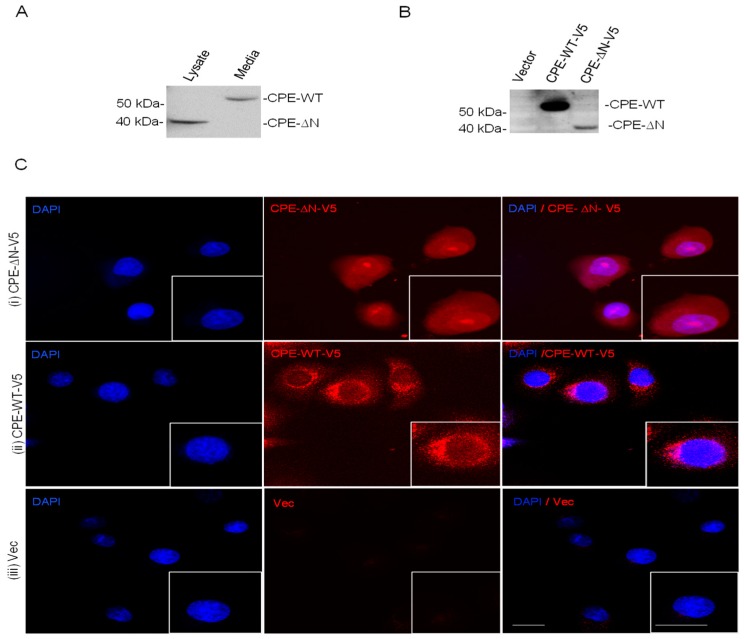
Cellular localization of CPE-ΔN and CPE-WT in Panc-1 cells. (**A**) Western blot showing CPE-WT in the concentrated medium of the Panc-1 cells but not in the cell lysate. Note, CPE-ΔN was found in the cell lysates but not in the secretion medium. (**B**) Western blot showing expression of CPE-WT-V5, CPE-ΔN-V5, and vector (Vec) alone in the stably transfected Panc-1 cells using anti-V5 antibody. Full-length blots are presented in Supplementary Figure S2A,B (**C**) Representative confocal images of Panc-1 cells stably expressing CPE-WT-V5 and CPE-ΔN-V5 or vector alone (control). Anti-V5 (red) and nuclear staining with DAPI (blue) are shown. Note V5 tag staining in the cells expressing CPE-ΔN-V5 was localized in the nucleus overlapping with DAPI stain, as well as in the cytoplasm (top panels) while V5 staining in cells expressing CPE-WT-V5 was localized exclusively in the cytoplasm (middle panels). Panc-1 cells expressing vector alone show only background staining for V5 since this tag is not in the construct (lower panels). Inset shows high magnification image of a single cell. Scale bar = 20 μm.

Analysis of the cell lysate and concentrated culture media of Panc-1 cells showed the presence of 53 kDa CPE in the media but not CPE-ΔN. In contrast, the cell lysate showed only 40 kDa CPE-ΔN protein (Figure 2A). These data indicated that endogenous CPE-WT protein was secreted, while the 40 kDa CPE-ΔN remained within the cells. To study the subcellular localization of CPE-WT and CPE-ΔN, we carried out immunocytochemistry on the Panc-1 cells stably transfected with V5 tagged CPE-WT and CPE-ΔN. The expression of the V5 tag was validated by Western blot, which showed V5 bands corresponding to the size of CPE-WT and CPE-ΔN in these transfected Panc-1 cells (Figure 2B). Immunocytochemical analysis revealed that CPE-ΔN-V5 immunostaining overlapped with DAPI (4,6-Diamidino-2-phenylindole) staining, indicating its localization in the nucleus, as well as in the cytoplasm (Figure 2Ci). However, in CPE-WT-V5 transfected Panc-1 cells, V5 immunostaining was not present in the nucleus, but only in the cytoplasm, showing a punctate appearance indicative of the presence in secretory vesicles (Figure 2Cii). As a negative control, cells transfected with the vector alone showed no V5 immunostaining (Figure 2Ciii). These results indicated that CPE-ΔN, but not CPE-WT was localized in the nucleus of the Panc-1 cells.

### 2.3. Overexpression of CPE-ΔN in Panc-1 Cells Increases Proliferation and Invasion

**Figure 3:**
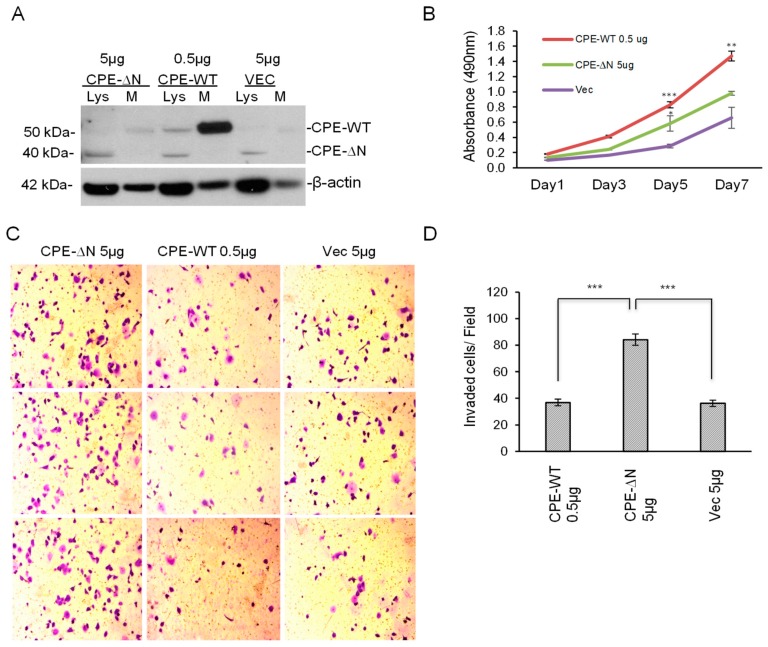
CPE-ΔN enhances proliferation and invasion of Panc-1 cells. (**A**) Western blot analysis of cell lysates (Lys) or secretion media (M) of Panc-1 cells transfected with 5 µg or 0.5 µg of the plasmid (as indicated) carrying construct for 40 kDa CPE-ΔN, CPE-WT or vector control for 24 h. β-actin was used as the loading control for the cellular lysates. Note that β-actin bands in the media fraction cannot be considered as a loading control and is possibly the result of minimal cell lysis before supernatant media collection. Full-length blots are presented in Supplementary Figure S2C,D. (**B**) A line graph of an MTT assay showing proliferation of Panc-1 cells expressing 40 kDa CPE-ΔN, CPE-WT, or vector control (Vec) from Day 1–5. (*N* = 1, *n* = 3, ** *p* < 0.01 *** *p* < 0.001). (**C**) Representative images of Panc-1 cells in Matrigel invasion assay 24 h after transfection with 40 kDa CPE-ΔN, CPE-WT, or vector control. (**D**) Bar graph showing increased invasion of Panc-1 cells expressing 40 kDa CPE-ΔN compared to vector control (*N* = 1, *n* = 3, *** *p* < 0.001). Error bars denote SD.

The effect of overexpression of 40 kDa CPE-ΔN in promoting proliferation and invasion of Panc-1 cells was investigated. Since the expression of exogenous CPE-WT protein was at a much higher level (at least 10 times higher) than CPE-ΔN, as shown in Figure 2B, in order to compare the efficacy of CPE-WT with CPE-ΔN, we transfected Panc-1 cells with CPE-WT and CPE-ΔN constructs at different plasmid concentrations, such that the expression levels of each of these proteins in the cell lysate were comparable. In the case of CPE-WT, more of the protein was present in the medium than in the cell lysate (Figure 3A). Analysis using the MTT assay showed that Panc-1 cells overexpressing CPE-ΔN exhibited ~1.5-fold enhanced proliferation after five days compared to the control cells transfected with the vector alone. CPE-WT transfected cells demonstrated a ~2.2-fold increase in proliferation compared to empty vector control cells at day 5 (Figure 3B). In the Matrigel invasion assay, Panc-1 cells overexpressing CPE-ΔN displayed significantly enhanced invasion (~2.3-fold) compared to Panc-1 cells transfected with an empty vector, whereas cells overexpressing CPE-WT showed similar invasion to the control cells (Figure 3C,D).

### 2.4. CPE-ΔN Upregulates CXCR2 Expression in Panc-1 Cells

**Figure 4:**
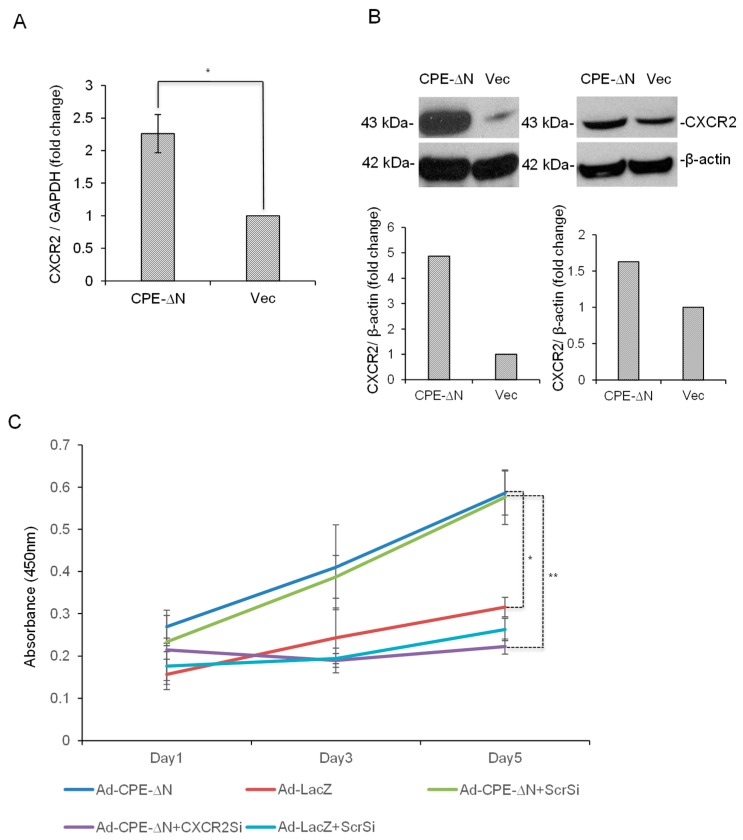
CPE-ΔN upregulates CXCR2 expression and promotes the proliferation of Panc-1 cells in a CXCR2-dependent manner. (**A**) Bar graph showing the increase in expression of *CXCR2* mRNA in Panc-1 cells transfected with 2 µg of CPE-ΔN plasmid compared to control cells. GAPDH (glyceraldehyde 3-phosphate dehydrogenase) was used as the reference gene for qRT-PCR analysis. (*N* = 2, *n* = 3, * *p* < 0.05). Error bars denote SEM. (**B**) Western blot images (upper panel) and the corresponding quantitative analysis (lower panel) showing an increase in expression of CXCR2 protein in Panc-1 cells transfected with 3 µg of CPE-ΔN compared to vector-transfected cells. β-actin was used as the loading control for Western blot. Full-length blots are presented in Supplementary Figure S3. (**C**) Line graph of MTT assay showing the proliferation of Panc-1 cells transduced with adenovirus carrying CPE-ΔN for 48 h followed by transfection (for 48 h) with CXCR2 siRNA on day 1, day 3 and day 5. Adenovirus encoding LacZ and scrambled siRNA were used as controls for CPE-ΔN overexpression and transfection, respectively. (*N* = 1, *n* = 3, * *p* < 0.05, ** *p* < 0.01). Error bars denote SD.

As CXCR2 expression was upregulated downstream of CPE-ΔN in the HCC cells [19], we speculated whether CXCR2, a chemokine receptor implicated in driving pancreatic cancer metastasis [17] could be regulated by CPE-ΔN in Panc-1 cells as a mechanism to promote the proliferation and invasion of these cells. We found that Panc-1 cells transfected with the CPE-ΔN construct for 48 h showed a ~2.2-fold increase in expression of *CXCR2* mRNA (Figure 4A) and a marked increase in the CXCR2 protein (Figure 4B), while transfection of CPE-WT only marginally enhanced *CXCR2* mRNA and protein levels (Supplementary Figure S4).

### 2.5. CPE-ΔN Induces Proliferation of Panc-1 Cells in a CXCR2-Dependent Manner

Next, we determined if the enhancement of proliferation by CPE-ΔN in Panc-1 cells was associated with the downstream expression of CXCR2. Indeed, on day 5 of the MTT assay, we observed a ~2.5-fold decrease in the proliferation of Panc-1 cells overexpressing CPE-ΔN following the suppression of CXCR2 levels by RNAi, when compared to similar cells treated with scrambled siRNA (Figure 4C). These data suggest that CXCR2 mediates the tumorigenic functions of CPE-ΔN during pancreatic cancer progression.

## 3. Discussion

Most pancreatic cancers are diagnosed at advanced disease stages when the tumor has metastasized, leading to poor prognosis [23]. Elucidating molecules that contribute to the growth and metastasis of these cancers will facilitate the finding of therapeutic agents that can disrupt this process. In this study, we have demonstrated that CPE, especially a 40 kDa CPE-ΔN isoform was important in promoting proliferation and invasion of pancreatic cancer cells. Northern blot analysis detected two CPE transcripts of 2.4 kb and 1.7 kb in size encoding CPE-WT and a 40 kDa N-terminal truncated CPE variant, CPE-ΔN, respectively, in BXPC-3 pancreatic cancer cells, but only the 2.4 kb transcript was detectable in Panc-1, likely due to the low abundance or rapid turnover of the 1.7 kb transcript. Nevertheless, Western blot analysis showed expression of 40 kDa CPE-ΔN protein in Panc-1 cells, as well as some CPE-WT in the secretion medium. BXPC-3 expressed both CPE-WT and 40 kDa CPE-ΔN protein. Pull-down experiments indicated that 40 kDa CPE-ΔN protein was recognized by two different antibodies. Analysis of the cell lysate and secretion media of Panc-1 cells suggested a low level of expression of CPE-WT protein in these cells relative to the large amounts of CPE mRNA present in the cells. Subcellular localization studies showed endogenous and transfected 40 kDa CPE-ΔN in the nucleus as revealed by Western blot and immunocytochemistry. The nuclear localization of CPE-ΔN in the HCC cells was consistent with its role in regulating the expression of several metastasis-related genes in these cells [19].

Gain-of-function studies determined the efficacy of CPE-WT versus CPE-ΔN in promoting proliferation and invasion. A previous study showed that knockdown of endogenous CPE levels in BXPC-3 suppressed the proliferation, migration, and tumor-forming ability of these cells while showing improved sensitivity to the chemotherapeutic drug, cisplatin. Increased expression of CPE was found in tumor tissues from four pancreatic cancer patients. The study identified NF-κB as the probable downstream target through which CPE exerted its effects on pancreatic cancer [22]. Since the CPE siRNA used in this study knocks down both WT and the splice variant, it was difficult to attribute the functional deficits to either CPE-WT and/or CPE-ΔN. Hence, we performed gain-of- function studies in Panc-1 cells, which expressed relatively low amounts of CPE compared to BXPC-3 (Figure 1), in order to distinguish the effects of CPE-WT and CPE-ΔN in promoting PC growth and invasion. Our data indicated that the overexpression of approximately equivalent amounts of CPE-WT and CPE-ΔN protein in Panc-1 cells, both significantly enhanced the proliferation of these cancer cells. However, while Panc-1 cells transfected with CPE-ΔN exhibited a significant increase in invasion, cells transfected with CPE-WT expressed at a similar or even a higher level to CPE-ΔN, if the secreted amounts were considered, and showed no increase in invasion compared to the control cells. Thus, CPE-ΔN is an important player in promoting growth and invasion of pancreatic cancer cells.

Chemokines have been reported to contribute to the progression and metastasis of pancreatic cancer, especially pancreatic ductal adenocarcinoma (PDAC) [24]. Angiogenic CXC chemokines acting through the CXCR2 receptor are known to interact with myeloid cells in the tumor microenvironment and influence the cancer outcome [17,18]. CXCR2 signaling also acts downstream of the *KRAS* mutation or possibly in a feed-forward loop to drive the development of PDAC [16]. Besides pancreatic cancer, CXCR2 promotes tumorigenesis and metastasis in lung, breast, colon, and skin cancers [18,25]. Our recent study showed that CPE-ΔN can enhance *CXCR2* mRNA levels in HCC cells [19], which was the rationale for investigating if CXCR2 mediated the downstream effects of CPE-ΔN on Panc-1 cells. We demonstrated that Panc-1 cells transiently transfected with CPE-ΔN significantly enhanced expression of CXCR2 compared to control cells, and that CPE-ΔN induced an increase in proliferation of Panc-1 cells, which was mediated by CXCR2. Hence, CPE-ΔN may promote growth and invasion of PC cells, via CXCR2, and possibly in conjunction with other metastatic genes such as MMP3 and CCL12, found to be upregulated by CPE-ΔN in HCC cells [19]. More studies, especially using in vivo models, are warranted to examine the mechanism of how CPE-ΔN regulates CXCR2.

EMT (epithelial–mesenchymal transition) is thought to be the underlying mechanism that promotes metastatic dissemination and chemotherapeutic resistance associated with PC [26,27]. EMT occurs as an early event in a pre-metastatic lesion or PanIN during PC progression [27,28]. It is characterized by the downregulation of E-cadherin and miR200 levels, and activation of TGF-b signaling to increase the expression of transcriptional factors that regulate EMT such as ZEB1, Snail and Slug [29,30]. A recent study demonstrated that CPE-ΔN enhanced the migration and invasion of human OS cells while promoting their epithelial–mesenchymal transition by activation of the Wnt/ β-catenin pathway [31]. Stable CPE-ΔN expressing OS cell lines were used to demonstrate that CPE-ΔN significantly downregulated E-cadherin expression while upregulated vimentin and transcription factors snail and slug, thus facilitating the EMT process in OS cells. It would be interesting to determine if CPE-ΔN supports the EMT process in PDAC development, based on our data that it augments the invasive property of PC cells. Nearly 94% of PDAC have *KRAS* mutations, which drive the initiation of PanIN formation and subsequent development to malignant disease [32,33]. Besides PC, *KRAS* mutations are predominant in lung and colorectal cancers [34]. A few reports suggest that CPE/ CPE-ΔN could be involved in promoting these cancers. One study investigated if CPE-∆N protein expression is valuable in early identification of recurring and metastatic lung adenocarcinoma. Out of the 95 patient samples that were analyzed for protein expression of CPE-WT and CPE-∆N using Western Blot and IHC, it was observed that while WT-CPE was expressed at similar levels in tumor tissue and peri-carcinoma tissues, CPE-∆N was prominently expressed only in the tumor tissue. Multivariate Cox regression analysis revealed that CPE-∆N expression was associated with lymph node and distant metastasis. Patients having CPE-∆N expression exhibited higher 3-year postoperative recurrence and metastasis rates than patients lacking CPE-∆N expression [20]. Enhanced CPE expression was documented in colorectal cancer (CRC) cell lines and patient tumor samples. Using two CRC cell lines, it was shown that overexpression of full-length CPE enhanced the proliferation and colony-forming ability of these cells, by increasing the percentage of cells in the S-phase of cell cycle, as determined by flow cytometry. In concurrence, CPE down-regulated the expression of CDK inhibitors p21 and p27 and increased cyclin D1 expression [8]. Hence, CPE-ΔN and CPE-WT contribute to tumor progression and invasion, possibly by distinct mechanisms depending on the cancer type.

Considering the importance of CPE-ΔN in regulating the growth and invasion of PC cells, it could be an effective therapeutic strategy to target CPE-ΔN. However, there are some challenges. RNAi mediated suppression of CPE is not specific to CPE-ΔN, and can affect CPE-WT as well. Designing specific inhibitors for CPE-ΔN requires further understanding of how the synthesis of CPE-ΔN is regulated at the transcriptional and epigenetic levels. Moreover, the mechanism of regulation of target genes by CPE-ΔN remains unclear. Future research centered on addressing these questions will provide clues to achieve specific targeting of CPE-ΔN. We propose that CPE-ΔN inhibition could be used as a combination therapy to anti-KRAS therapy against PC. In conclusion, 40 kDa CPE-ΔN has a powerful effect on promoting proliferation and invasion of Panc-1 cells, partly through the upregulation of the CXCR2 chemokine receptor, known to drive PDAC progression and metastasis. Thus, 40 kDa CPE-ΔN is an excellent therapeutic target for the treatment of invasive cancer of the pancreas.

## 4. Materials and Methods

### 4.1. Cell Lines

Human pancreatic cell lines Panc-1 and BXPC-3 were obtained from ATCC (Manassas, VA, USA). The cells were cultured in a DMEM media (Millipore Sigma, Burlington, MA, USA), supplemented with 10% fetal bovine serum (Thermo Fisher Scientific, Waltham, MA, USA) at 37 °C in a humidified 5% CO_2_ incubator.

### 4.2. Northern Blot

Northern blot analysis was performed using a Northern Max kit (Ambion- Thermo Fisher Scientific) according to the manufacturer’s instructions. Briefly, a 188 bp human DIG (digoxigenin)-labeled (DIG RNA labeling kit, Roche, Mannheim, Germany) CPE probe covering the middle region (1241–1428 nt) of human CPE mRNA (GenBank accession number: NM_001873.2) was used. Messenger RNA samples (2.5 µg) were run on a denaturing formaldehyde gel and blotted to a nylon membrane. Membranes were hybridized overnight at 50 °C, washed and processed for immunodetection using an anti-DIG-AP antibody (1:10,000, Roche) and visualized by CSPD [Disodium 3-(4-methoxyspiro {1,2-dioxetane-3,2′-(5′-chloro)tricyclo [3.3.1.1]decan}-4-yl)phenyl phosphate] chemiluminescence substrate (1:100, Roche) against X-film. 

### 4.3. Transfection of Cells with CPE-ΔN and CPE-WT Constructs

Plasmid constructs containing *CPE-WT* or *CPE-ΔN* sequences were generated with a pcDNA3.1 vector (Invitrogen, Carlsbad, CA, USA), and a V5-Tag was inserted into the C-terminal of both clones [19]. Transfection was performed using a Lipofectamine^®^ 2000 reagent (Invitrogen) according to the manufacturer’s protocol (Invitrogen). Briefly, Panc-1 cells were transiently transfected with different doses of plasmids carrying *CPE-ΔN* (5 µg) or *CPE-WT* (0.5 µg) or as mentioned in the Figure legends. For stable transfection, 4 µg of plasmids were used, and after 48 h, a selection medium containing 800 µg/mL of Geneticin (GIBCO- Life Technologies, Carlsbad, CA, USA) was added to the transfected cells. The cells were maintained in the selection media for about 2 weeks, to enrich for stably transfected clones. Stable integration of the plasmid in the cell line was confirmed by Western blot analysis.

### 4.4. Immunoprecipitation of CPE/CPE-ΔN

Cell extract (~1 mg protein) prepared in TNE buffer (Tris 50 mM, NaCl 150 mM, EDTA 5 mM, pH 7.4) containing 1% NP-40 and 1X protease inhibitor cocktail, were incubated with rabbit polyclonal anti-CPE antibody 6135 (7µg, generated in our laboratory) or control rabbit IgG (7 µg, Invitrogen) overnight at 4 °C, followed by incubation with Protein A/G PLUS-Agarose beads (Santa Cruz Biotechnology, Dallas, TX, USA) for 2 h. After thorough washing with lysis buffer to remove the non-specific binding, the protein-antibody complex was eluted from the beads by boiling in SDS (sodium dodecyl sulfate) protein gel loading solution (Quality Biological, Gaithersburg, MD, USA). Equal volumes of eluate were analyzed by Western blot using anti-CPE mouse monoclonal antibody (BD Biosciences, Franklin Lakes, NJ, USA).

### 4.5. Western Blot

Cells were extracted using a RIPA (radioimmunoprecipitation assay) buffer (Thermo Fisher Scientific) supplemented with Complete Inhibitor Cocktail (Roche Applied Science, Indianapolis, IN). For secretion protein analysis, ~2.5 × 10^6^ cells were seeded in a T25 flask and grown to ~90% confluency. The supernatant media were replaced with serum-free DMEM for 18 h (4 mL) and then collected and concentrated to ~200 µL using an Amicon Ultra 10k MWCO centrifugal filter (Millipore Sigma, Burlington, MA, USA). Protein (~25 µg) from cell lysate or media concentrate was loaded on the SDS-PAGE gels and analyzed by Western blotting, as previously described [7]. CPE-WT and CPE-ΔN were detected using monoclonal antibody (1:5000 dilution, BD Biosciences). Anti-CXCR2 antibody (1:500 dilution) was from Abcam (Cambridge, MA, USA), and β-actin (1:5000 dilution) was from Cell Signaling Technology (Danvers, MA, USA).

### 4.6. Immunocytochemistry

Panc-1 cells stably expressing CPE-WT/ 40 kDa CPE-ΔN or vector alone were processed for immunocytochemistry (ICC) 24 h after seeding. Cells were incubated with anti-V5 (1:400 dilution, Invitrogen) primary antibody overnight at 4 °C and then with Goat IgG Alexa 568 (red) as a secondary antibody (1:1000 dilution, Invitrogen) for 1h at room temperature. DAPI (4,6-Diamidino-2-phenylindole) at 1:50,000 dilution was used for staining DNA. Fluorescent images of V5 staining were captured with a Zeiss LSM510 confocal microscope (Carl-Zeiss, San Diego, CA, USA).

### 4.7. Quantitative Real-Time RT-PCR

RNA was extracted from Panc-1 cells using the TRIzol reagent (Sigma-Aldrich, St. Louis, MO, USA) according to the manufacturer’s instructions, and the first-strand cDNA was synthesized with 5 μg of total RNA using the SensiFAST cDNA Synthesis Kit (Bioline Meridian Life Science, Inc, Memphis, TN, USA). For CXCR2 expression analysis, quantitative PCR was performed using SYBR Green Master Mix, as previously described (Invitrogen) [7]. GAPDH was used for normalizing CXCR2. Primer sequences were: *GAPDH*, fwd: 5′–ACCACAGTCCATGCCATCAC and rev: 5′–TCCACCACCCTGTTGCTGTA; *CXCR2*, fwd: 5′–CATGGCTTGATCAGCAAGGA and rev: 5′–TGGAAGTGTGCCCTGAAGAAG.

### 4.8. MTT Cell Proliferation Assay

Panc-1 cells were seeded in a 96-well plate at a density of 2000 cells/well. An MTT (3-(4,5-dimethylthiazol-2-yl)-2,5-diphenyltetrazolium bromide) assay was performed from days 1–4 or at 5 days, as reported previously [35]. Briefly, 25 µL of MTT reagent (5 mg/mL) (Sigma-Aldrich) was added to each well and incubated for 4 h at 37 °C in a CO_2_ incubator. The supernatant was removed after incubation, and 150 µL of DMSO was added to each well. Five minutes later, the absorbance was measured at 490 nm or 450 nm in a microplate reader (BioTek, Winooski, VT, USA).

### 4.9. Invasion Assay

A cell invasion assay was performed in a 24-well Corning Matrigel invasion chamber (Corning, NY, USA) with 8 μm pores. Briefly, cell suspension containing 1 × 10^5^ cells/ ml in serum-free media was added to the top chamber and serum-supplemented media to the lower chamber. After 24 h, cells that failed to invade through the pores were carefully removed using a cotton swab. The cells on the lower surface of the membrane were fixed with 100% methanol and stained with 1% crystal violet solution for 10 min. After removing the excess stain with water, images from 5 different fields within the well were captured, and the cells were counted.

### 4.10. Statistical Analysis

Data represent the mean of at least triplicate values (*n*) from independent experiments (*N*) as indicated in the figure legends. Significance was determined by a Student’s t-test and p values are denoted as * *p* < 0.05, ** p < 0.01, *** *p* < 0.001. Error bars denote standard deviation (SD) or standard error of the mean (SEM).

## Supplementary Materials

Supplementary materials can be found at https://www.mdpi.com/1422-0067/20/22/5725/s1.

## Abbreviations

CPE	Carboxypeptidase E
PC	Pancreatic cancer
HCC	Hepatocellular carcinoma
WT	Wild-type
PDAC	Pancreatic ductal adenocarcinoma
OS	Osteosarcoma

## References

[1] Khan M.A., Azim S., Zubair H., Bhardwaj A., Patel G.K., Khushman M., Singh S., Singh A.P. (2017). Molecular Drivers of Pancreatic Cancer Pathogenesis: Looking Inward to Move Forward. Int. J. Mol. Sci..

[2] Hines O.J., Reber H.A. (2008). Pancreatic surgery. Curr. Opin. Gastroenterol..

[3] Cheng Y., Cawley N.X., Loh Y.P. (2014). Carboxypeptidase E (NF-alpha1): A new trophic factor in neuroprotection. Neurosci. Bull..

[4] Selvaraj P., Xiao L., Lee C., Murthy S.R., Cawley N.X., Lane M., Merchenthaler I., Ahn S., Loh Y.P. (2017). Neurotrophic Factor-alpha1: A Key Wnt-beta-Catenin Dependent Anti-Proliferation Factor and ERK-Sox9 Activated Inducer of Embryonic Neural Stem Cell Differentiation to Astrocytes in Neurodevelopment. Stem Cells.

[5] Armento A., Ilina E.I., Kaoma T., Muller A., Vallar L., Niclou S.P., Kruger M.A., Mittelbronn M., Naumann U. (2017). Carboxypeptidase E transmits its anti-migratory function in glioma cells via transcriptional regulation of cell architecture and motility regulating factors. Int. J. Oncol..

[6] Cawley N.X., Wetsel W.C., Murthy S.R., Park J.J., Pacak K., Loh Y.P. (2012). New roles of carboxypeptidase E in endocrine and neural function and cancer. Endocr. Rev..

[7] Murthy S.R.K., Dupart E., Al-Sweel N., Chen A., Cawley N.X., Loh Y.P. (2013). Carboxypeptidase E promotes cancer cell survival, but inhibits migration and invasion. Cancer Lett..

[8] Liang X.H., Li L.L., Wu G.G., Xie Y.C., Zhang G.X., Chen W., Yang H.F., Liu Q.L., Li W.H., He W.G. (2013). Upregulation of CPE promotes cell proliferation and tumorigenicity in colorectal cancer. BMC Cancer.

[9] Huang S.F., Wu H.D., Chen Y.T., Murthy S.R., Chiu Y.T., Chang Y., Chang I.C., Yang X., Loh Y.P. (2016). Carboxypeptidase E is a prediction marker for tumor recurrence in early-stage hepatocellular carcinoma. Tumour Biol..

[10] Shen H.W., Tan J.F., Shang J.H., Hou M.Z., Liu J., He L., Yao S.Z., He S.Y. (2016). CPE overexpression is correlated with pelvic lymph node metastasis and poor prognosis in patients with early-stage cervical cancer. Arch. Gynecol. Obstet..

[11] Wang X.C., Xu S.Y., Wu X.Y., Song H.D., Mao Y.F., Fan H.Y., Yu F., Mou B., Gu Y.Y., Xu L.Q. (2004). Gene expression profiling in human insulinoma tissue: Genes involved in the insulin secretion pathway and cloning of novel full-length cDNAs. Endocr. Relat. Cancer..

[12] He P., Varticovski L., Bowman E.D., Fukuoka J., Welsh J.A., Miura K., Jen J., Gabrielson E., Brambilla E., Travis W.D. (2004). Identification of carboxypeptidase E and gamma-glutamyl hydrolase as biomarkers for pulmonary neuroendocrine tumors by cDNA microarray. Hum. Pathol..

[13] Grimwood B.G., Plummer T.H., Tarentino A.L., Carboxypeptidase H. (1989). A regulatory peptide-processing enzyme produced by human hepatoma Hep G2 cells. J. Biol. Chem..

[14] Manser E., Fernandez D., Lim L. (1991). Processing and secretion of human carboxypeptidase E by C6 glioma cells. Biochem. J..

[15] Horing E., Harter P.N., Seznec J., Schittenhelm J., Buhring H.J., Bhattacharyya S., von Hattingen E., Zachskorn C., Mittelbronn M., Naumann U. (2012). The “go or grow” potential of gliomas is linked to the neuropeptide processing enzyme carboxypeptidase E and mediated by metabolic stress. Acta Neuropathol..

[16] Purohit A., Varney M., Rachagani S., Ouellette M.M., Batra S.K., Singh R.K. (2016). CXCR2 signaling regulates KRAS(G(1)(2)D)—Induced autocrine growth of pancreatic cancer. Oncotarget.

[17] Steele C.W., Karim S.A., Leach J.D.G., Bailey P., Upstill-Goddard R., Rishi L., Foth M., Bryson S., McDaid K., Wilson Z. (2016). CXCR2 Inhibition Profoundly Suppresses Metastases and Augments Immunotherapy in Pancreatic Ductal Adenocarcinoma. Cancer Cell.

[18] Yang L., Huang J., Ren X., Gorska A.E., Chytil A., Aakre M., Carbone D.P., Matrisian L.M., Richmond A., Lin P.C. (2008). Abrogation of TGF beta signaling in mammary carcinomas recruits Gr-1+CD11b+ myeloid cells that promote metastasis. Cancer Cell.

[19] Yang X., Lou L., Chen Y., Huang S., Loh Y.P. (2019). A novel 40kDa N-terminal truncated Carboxypeptidase E splice variant: Cloning, cDNA sequence analysis and role in regulation of metastatic genes in human cancers. Genes Cancer.

[20] Sun J., Meng D., Li L., Tian X., Jia Y., Wang H., Yu H., Sun T., Qu A., Shen H. (2016). N-terminal truncated carboxypeptidase E expression is associated with poor prognosis of lung adenocarcinoma. Oncol. Lett..

[21] Fan S., Li X., Li L., Wang L., Du Z., Yang Y., Zhao J., Li Y. (2016). Silencing of carboxypeptidase E inhibits cell proliferation, tumorigenicity, and metastasis of osteosarcoma cells. Onco Targets Ther..

[22] Liu A., Shao C., Jin G., Liu R., Hao J., Shao Z., Liu Q., Hu X. (2014). Downregulation of CPE regulates cell proliferation and chemosensitivity in pancreatic cancer. Tumour Biol..

[23] Oldfield L.E., Connor A.A., Gallinger S. (2017). Molecular Events in the Natural History of Pancreatic Cancer. Trends Cancer.

[24] Du Y., Zhao B., Liu Z., Ren X., Zhao W., Li Z., You L., Zhao Y. (2017). Molecular Subtyping of Pancreatic Cancer: Translating Genomics and Transcriptomics into the Clinic. J. Cancer.

[25] Jamieson T., Clarke M., Steele C.W., Samuel M.S., Neumann J., Jung A., Huels D., Olson M.F., Das S., Nibbs R.J. (2012). Inhibition of CXCR2 profoundly suppresses inflammation-driven and spontaneous tumorigenesis. J. Clin. Investig..

[26] Arumugam T., Ramachandran V., Fournier K.F., Wang H., Marquis L., Abbruzzese J.L., Gallick G.E., Logsdon C.D., McConkey D.J., Choi W. (2009). Epithelial to mesenchymal transition contributes to drug resistance in pancreatic cancer. Cancer Res..

[27] Gaianigo N., Melisi D., Carbone C. (2017). EMT and Treatment Resistance in Pancreatic Cancer. Cancers (Basel).

[28] Ying H., Dey P., Yao W., Kimmelman A.C., Draetta G.F., Maitra A., DePinho R.A. (2016). Genetics and biology of pancreatic ductal adenocarcinoma. Genes Dev..

[29] Melisi D., Ishiyama S., Sclabas G.M., Fleming J.B., Xia Q., Tortora G., Abbruzzese J.L., Chiao P.J. (2008). LY2109761, a novel transforming growth factor beta receptor type I and type II dual inhibitor, as a therapeutic approach to suppressing pancreatic cancer metastasis. Mol. Cancer Ther.

[30] Filios S.R., Xu G., Chen J., Hong K., Jing G., Shalev A. (2014). MicroRNA-200 is induced by thioredoxin-interacting protein and regulates Zeb1 protein signaling and beta cell apoptosis. J. Biol. Chem.

[31] Fan S., Gao X., Chen P., Li X. (2019). Carboxypeptidase E-DeltaN promotes migration, invasiveness, and epithelial-mesenchymal transition of human osteosarcoma cells via the Wnt-beta-catenin pathway. Biochem. Cell Biol..

[32] Waters A.M., Der C.J. (2018). KRAS: The Critical Driver and Therapeutic Target for Pancreatic Cancer. Cold Spring Harb. Perspect. Med..

[33] Kanda M., Matthaei H., Wu J., Hong S.M., Yu J., Borges M., Hruban R.H., Maitra A., Kinzler K., Vogelstein B. (2012). Presence of somatic mutations in most early-stage pancreatic intraepithelial neoplasia. Gastroenterology.

[34] Choi J., Cox A.D., Li J., McCready W., Ulanova M. (2014). Activation of innate immune responses by Haemophilus influenzae lipooligosaccharide. Clin. Vaccine Immunol..

[35] Xiao L., Yang X., Sharma V.K., Loh Y.P. (2019). Cloning, gene regulation, and neuronal proliferation functions of novel N-terminal-truncated carboxypeptidase E/neurotrophic factor-alphal variants in embryonic mouse brain. FASEB J..

