# Supplementary material for: Carboxypeptidase E-∆N Promotes Proliferation and Invasion of Pancreatic Cancer Cells via Upregulation of CXCR2 Gene Expression
Source: Int J Mol Sci. 2019 Nov 15;20(22):5725. doi: 10.3390/ijms20225725 (PMC6888591; doi:10.3390/ijms20225725)
Supplement: Supplementary file 1 [file ijms-20-05725-s001.zip › Suppl IJMS 11-03-19.pptx]

## Slide 1
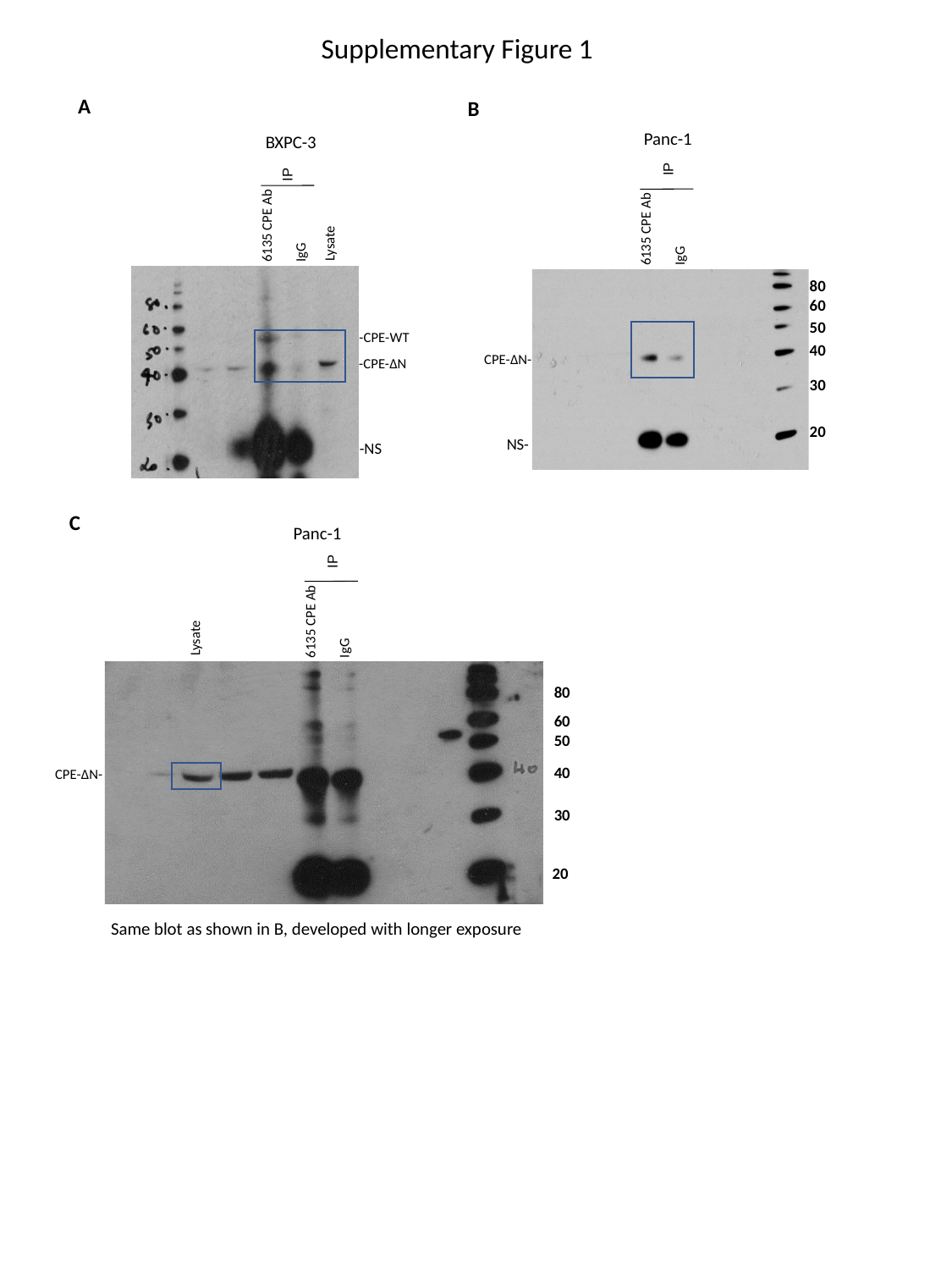

Supplementary Figure 1
A
B
 Panc-1
BXPC-3
IP
IP
6135 CPE Ab
6135 CPE Ab
Lysate
IgG
IgG
80
60
50
40
30
20
-CPE-WT
CPE-ΔN-
-CPE-ΔN
NS-
-NS
C
 Panc-1
IP
6135 CPE Ab
Lysate
IgG
80
60
50
40
30
20
CPE-ΔN-
Same blot as shown in B, developed with longer exposure

## Slide 2
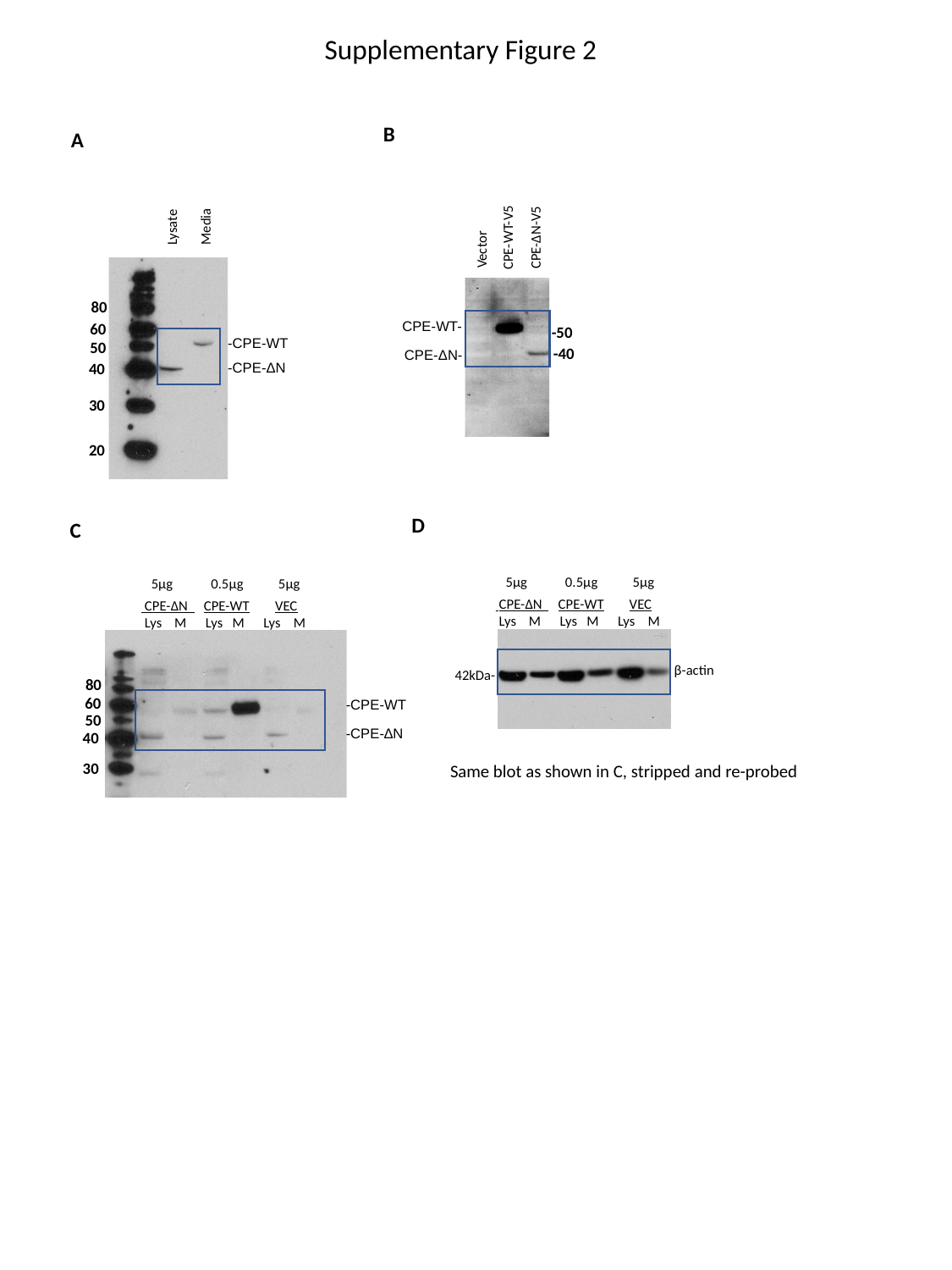

Supplementary Figure 2
B
A
Media
Lysate
Vector
CPE-WT-
-50
-40
CPE-ΔN-
CPE-ΔN-V5
CPE-WT-V5
80
60
-CPE-WT
50
40
-CPE-ΔN
30
20
D
C
5µg 0.5µg 5µg
 CPE-∆N CPE-WT VEC
 Lys M Lys M Lys M
β-actin
42kDa-
Same blot as shown in C, stripped and re-probed
5µg 0.5µg 5µg
 CPE-∆N CPE-WT VEC
 Lys M Lys M Lys M
80
60
-CPE-WT
50
-CPE-∆N
40
30

## Slide 3
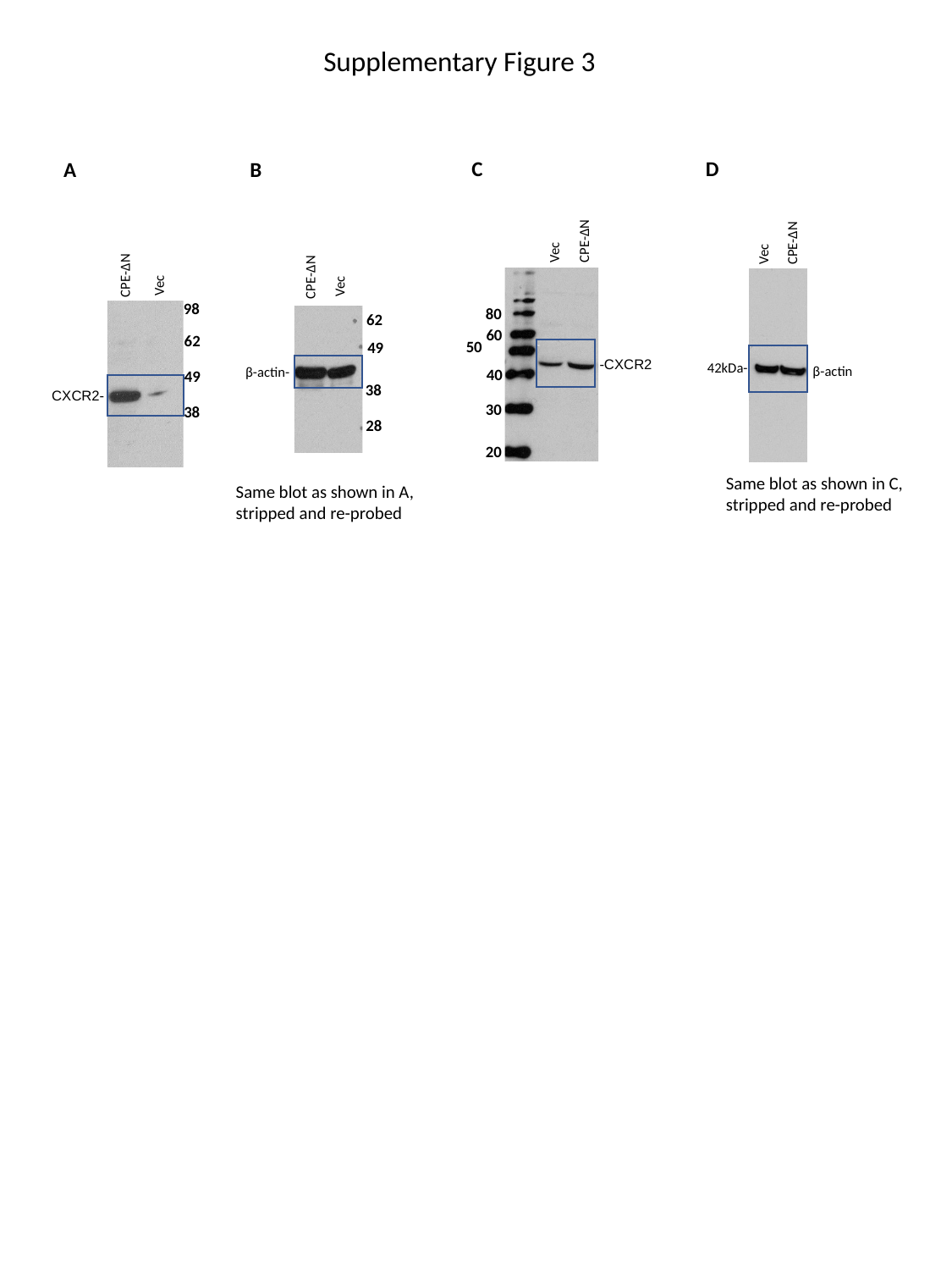

Supplementary Figure 3
C
D
B
A
CPE-ΔN
CPE-ΔN
CPE-∆N
Vec
62
50
49
38
28
Same blot as shown in A,
stripped and re-probed
Vec
Vec
CPE-∆N
Vec
98
62
49
CXCR2-
38
80
60
-CXCR2
42kDa-
β-actin
β-actin-
40
30
20
Same blot as shown in C,
stripped and re-probed

## Slide 4
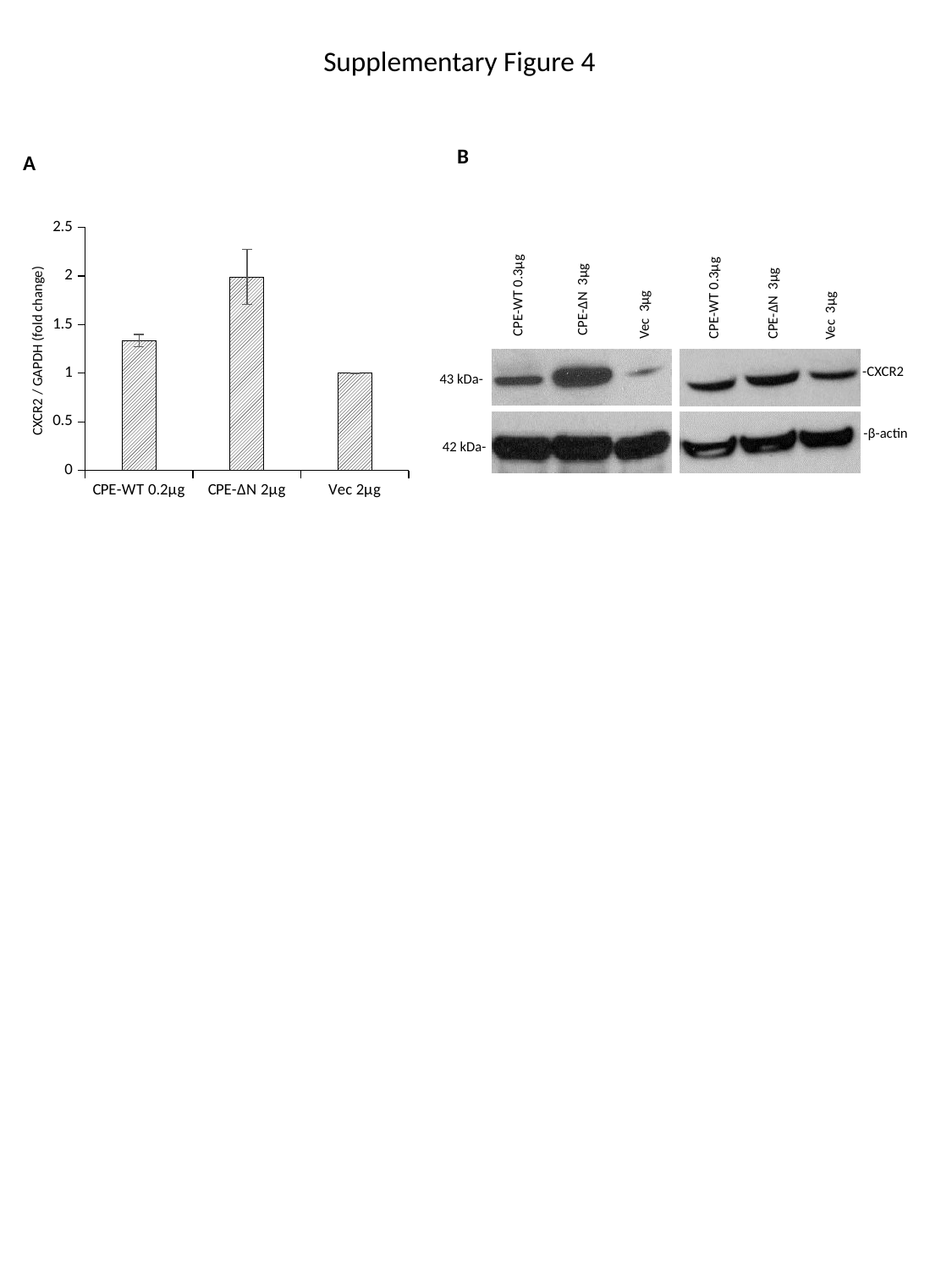

Supplementary Figure 4
B
A
### Chart
| Category | |
|---|---|
| CPE-WT 0.2µg | 1.335 |
| CPE-∆N 2µg | 1.99 |
| Vec 2µg | 1.0 |CPE-WT 0.3µg
CPE-WT 0.3µg
CPE-∆N 3µg
CPE-∆N 3µg
Vec 3µg
Vec 3µg
CXCR2 / GAPDH (fold change)
-CXCR2
43 kDa-
-β-actin
42 kDa-
