# Supplementary material for: Carboxypeptidase E-∆N Promotes Proliferation and Invasion of Pancreatic Cancer Cells via Upregulation of CXCR2 Gene Expression
Source: Int J Mol Sci. 2019 Nov 15;20(22):5725. doi: 10.3390/ijms20225725 (PMC6888591; doi:10.3390/ijms20225725)
Supplement: Supplementary file 1 [file ijms-20-05725-s001.zip › IJMS Suppl material Final 10-09-19.docx]

**Supplementary figure 4.** CPE proteins regulate CXCR2 expression in panc-1 cells. (**A).** Bar graph showing the increase in expression of *CXCR2* mRNA in Panc-1 cells transfected with 0.2µg of CPE-WT or 2µg of CPE-ΔN compared to control cells. GAPDH was used as the reference gene for qRT-PCR analysis. (N=1). Error bars denote SD. (**B**). Western blot images showing the increase in expression of CXCR2 protein in Panc-1 cells transfected with 0.3µg of CPE-WT or 3µg of CPE-ΔN compared to vector (3µg) transfected cells. β- actin was used as the loading control for Western blot.
